# Evaluation of fish feeder manufactured from local raw materials

**DOI:** 10.1038/s41598-021-98383-0

**Published:** 2021-09-22

**Authors:** El-Sayed Khater, Adel Bahnasawy, Osama Morsy

**Affiliations:** 1 Agricultural and Biosystems Engineering Department, Faculty of Agriculture, Benha University, Moshtohor, P.O. Box 13736, Toukh, Kalubia Egypt; 2 Basic and Applied Science Department, College of Engineering and Technology, Arab Academy for Science and Technology and Maritime Transport (AASTMT), P.O. Box 2033, Cairo, Egypt

**Keywords:** Ecology, Engineering

## Abstract

An automatic feeder for fish feeding was manufactured and evaluated successively. Feed pellet size, air flow rate and feeder screw speed were the most important factors affecting the performance and efficiency of the automatic feeder. It was tested at 3 sizes of pellets (1, 2 and 3 mm), 3 air flow rates (10, 15 and 20 m^3^ min^−1^) and 5 screw speeds (180, 360, 540, 720 and 900 rpm). The automatic feeder productivity, efficiency, specific energy consumption and costs were determined. The obtained results indicated that the automatic feeder productivity increases with increasing feed pellets size, air flow rate and rotational speed of screw treatments under study, the automatic feeder efficiency increased with increasing rotational speed of screw until it reached the highest value at 540 rpm and then remain constant at 720–900 rpm and after that decreased with increasing speed. Meanwhile, the specific energy consumption of automatic feeder decreased with increasing feed pellets size, air flow rate and rotational speed of screw treatments under study. The total cost of using automatic feeder ranged from 0.09 to 0.16 EGP kg^−1^ ($ = 15.63 EGP) for all treatments under study. This feeder will save time, effort and cost for fish industry.

## Introduction

Historical production data for aquaculture together with agricultural production data, show that aquaculture has been the fastest growing food production sector in the world, over the last few decades. Since 1984, global aquaculture output has increased at an average annual rate of about 10 percent, compared with a 3 percent increase for livestock meat and a 1.6 percent increase for capture fisheries. Aquaculture provided 8 percent of global fishery production (11 percent of food fish) in 1984, increasing to 22 percent (29 percent of food fish) in 1996, increasing to 51 percent (68 percent of food fish) in 2010 and increasing to 62 percent (75.5 percent of food fish) in 2019^1^.

Aquaculture generally needs more water per unit area or unit product than most other plant or animal production systems. Every kilogram of fish produced needs from 200 to 600 m^3^ of water. Some aquaculture system consume more water than other systems^2–4^. Aquaculture operations discharge large quantities of effluent which contains particulate and dissolved organic matter and nutrients. About 85% of phosphorus, 80–88% carbon and 52–95% of nitrogen inputs into fish culture system to be cost in the effluent water as uneaten feed, fish excretion, fecal production and respiration^5–7^.

In many developing countries, aquaculture activities (aquacultural facilitated required) are growing rapidly because it contributes in solving food security and poverty problems. A major challenge facing aquaculture development is the management of feeding systems. Feed control to meet fish needs is very important operation. Manual feeding operation is labor-intensive and expensive, which depends on many factors such as farm size, fish species and labor availability as well as feed frequency^8,9^.

Large catfish farms with several ponds needs to be fed only one time per day, on the other hands, small farms need more times a day. Generally, feed frequency increases with growth and feed conversion. In the intensive fish farming, fish needs to be fed many times a day in order to maximize the production at the optimum conditions. Feeding rate is affected also by the environmental conditions, fish weight and fish stock density^10,11^.

Feeding rates increases as the weight of fish increases but the increment percent decreases with increasing fish weight, where at early age of fish it is fed with 15% of its weight but at weights of above 200 g it is fed by 1–3% from its weight^12^. The optimal interval between feedings is suggested to be between 4 and 5 h which caused an increase in production and profit, this depends on the energy and composition of the diet^13^. A fish feeder was developed, fabricated and tested by Ref.^14^. It was fabricated from fiber reinforced plastic (FRP) material and used for crap feeding. Feeders controlled by fish needs and type^15^. Tadayoshi^16^ developed an automatic fish feeder which had the capability of sensing uneaten feed. Noor et al.^17^ designed an automatic fish feeder using PIC microcontroller. The basic components of the feeder are pellet storage, former, stand, DC motor and microcontroller.

Automatic feeders and feeding systems are playing a major role in the success of aquaculture farms globally and in the near future, it would become a necessity if intensive farming systems with more stocking density are to be adopted^18^. Fish feeding is a tedious and time-consuming operation, seeking for a tool to save effort and time beside of the uniformity distribution was the main aim of this work which is to manufacture a low cost automatic fish feeder which is made from local raw materials.

## Materials and methods

The main experiment was carried out at the workshop of Agricultural and Bio-Systems Engineering Department, Faculty of Agriculture Moshtohor, Benha University, Egypt to develop, fabricate and evaluate an automatic fish feeder.

## Materials

### Machine description

**Figure 1:**
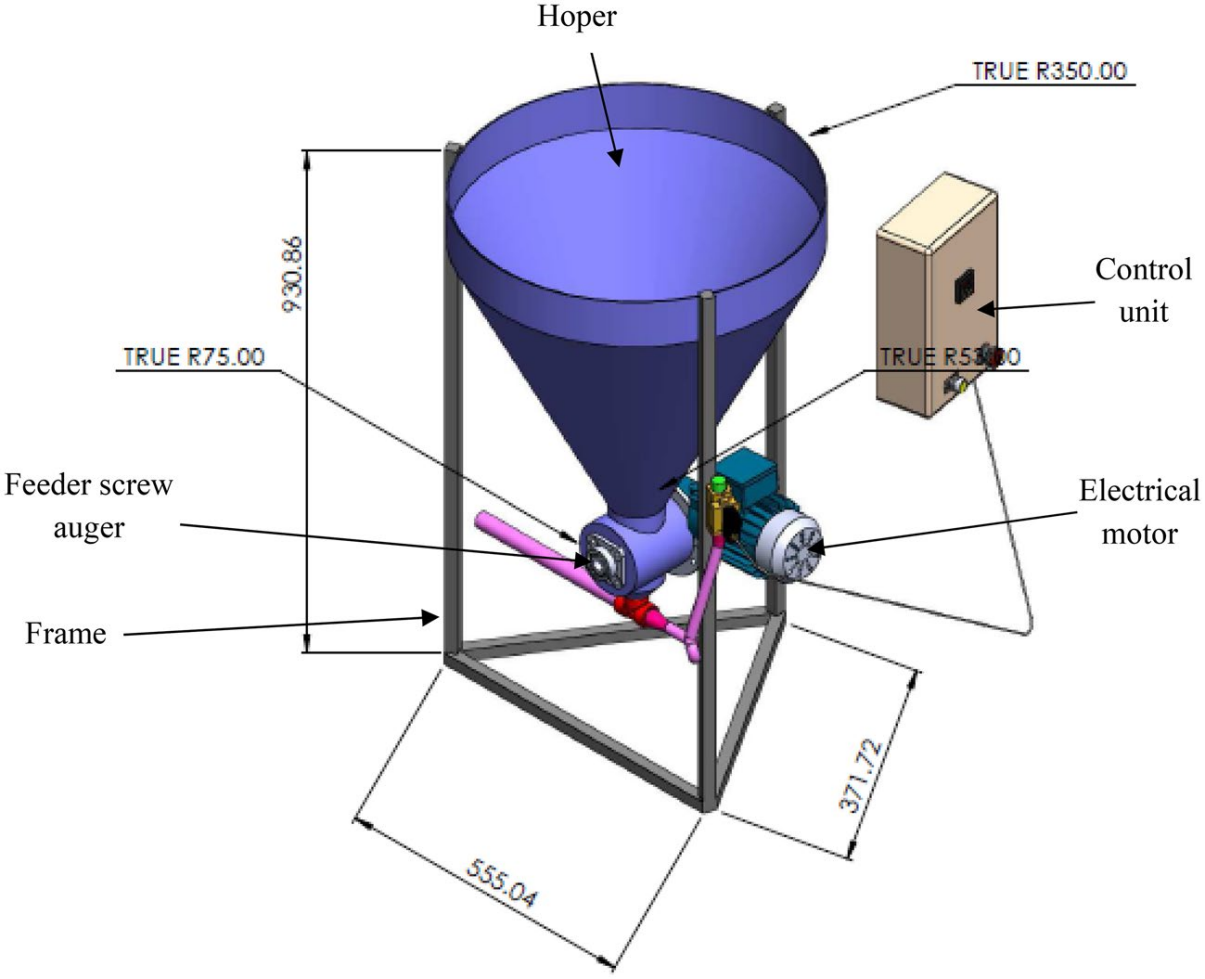
Isometric drawing of the automatic feeder (dimension in mm).

**Figure 2:**
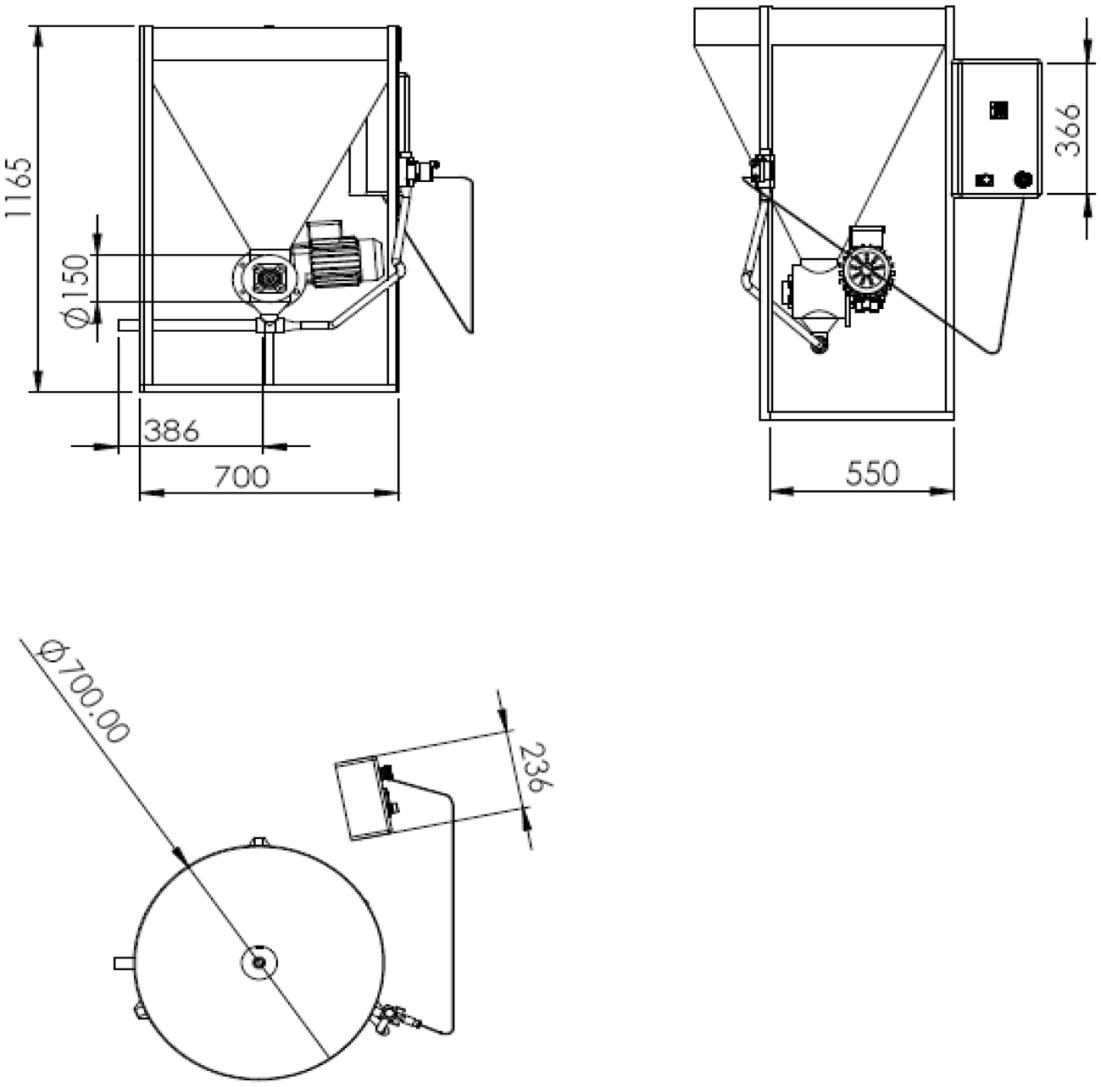
Orthographic drawing of the automatic fish feeder (dimension in mm).

The electrically operated machine was designed, fabricated and evaluated. Figures 1 and 2 show the isometric drawing, the orthographic drawing and the picture of the machine. The components of the machine include the machine frame, the feed hoper, screw auger, electrical motor and control unit.

### The machine frame

The main frame of the machine was constructed from steel box (30 × 30 × 3 mm for width, height and thickness, respectively). Dimensions of the machine frame are 700 mm length, 700 mm for width and 1165 mm for height.

### Feed hoper

The feed hopper is made of stainless steel (3 mm thickness) and it is a cylindrical shape. The top diameter is 70 cm, the under diameter is 15 cm and the height of feeder hoper is 70 cm. The capacity feed hopper was 90 L.

### Screw auger

**Figure 3:**
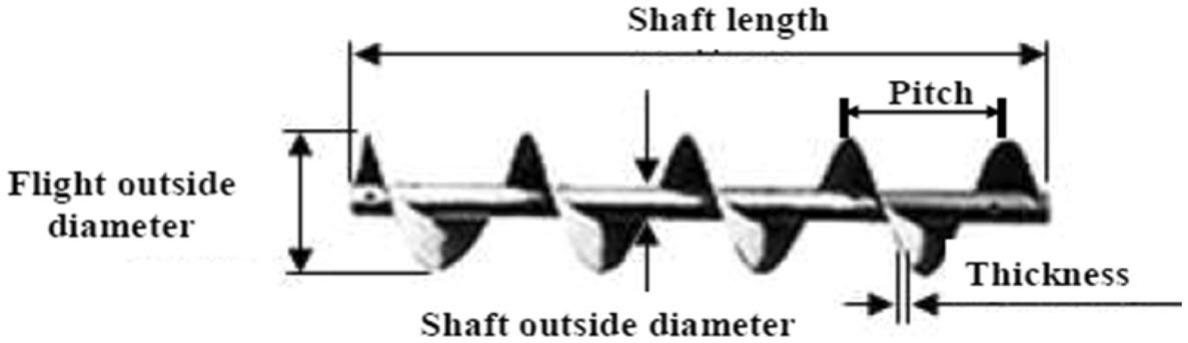
Components of screw auger.

Figure 3 shows the schematic diagram of the screw auger. The screw auger is made from local material and used to handle the product within the plant. Screw auger consists of a screw wraps around shaft. The shaft is fixed on a bearing from one side and connected to the motor directly.

### Electrical motor

The machine is driven by single phase electric motor (Model GAMAK—Power 0.37 kW 220 V 50 Hz, Turkey). The power was transmitted directly from the motor to main shaft of the screw auger, the maximum speed of motor is 1400 rpm.

### Control unit

Control unit was used to regulate the speed of motor from 1400 rpm to the required speeds to operate the auger screw. Also, control unit is used to control the operation time.

## Methods

The developed automatic fish feeder was evaluated by studying the effect of feed pellet size, air flow rate and rotational speed of screw on the productivity of automatic fish feeder.

### Experimental design

The treatments were arranged in a split-split plot design in three replications. The treatments include: three feed pellets sizes are 1, 2 and 3 mm, three air flow rates are 10, 15 and 15 m^3^ min^−1^ and five rotational speeds of screw are 180, 360, 540, 720 and 900 rpm with operating time of 5 min.

### Measurements

#### Automatic feeder productivity

The automatic feeder productivity (kg min^−1^) was determined as the amount of the fish feed during operation time.

#### Automatic feeder efficiency

Automatic feeder efficiency was estimated from equation:

1 $$ \eta = \frac{{\Pr_{actual} }}{{\Pr_{theoretical} }} \times {100,} $$

where η is the automatic feeder efficiency, %; Pr_actual_ is the actual productivity, kg min^−1^; Pr_theoretical_ is the theoretical productivity, kg min^−1^.

Theoretical productivity of automatic feeder was determined from equation:

2 $$ \Pr_{theoretical} = \frac{\pi }{4}{\text{PN}}\left( {{\text{D}}_{{1}}^{{2}} - D_{2}^{2} } \right)\rho {\text{Km,}} $$

where P is the pitch length, 3 cm; N is the rotational speed of screw, rpm; D_1_ is the flight outside diameter, 8 cm; D_2_ is the shaft outside diameter, 5 cm; ρ is the bulk density of fish feeds with different sizes, 6 × 10^–4^ kg cm^−3^; K is the loading factor, 0.55 based on repose angle of feeding raw materials; m is the inclination correction factor, 1 based on inclination angle of the augar.

### Power and energy requirement for automatic fish feeder

The power requirement (kW) was estimated by using the clamp meter to measure the line current strength (I) and the potential difference value (V).

The total electric power requirement under machine working load (P) was calculated according to Ref.^19^ by the following equation:

3 $$ P = \frac{I \times V \times \cos \theta }{{1000}}{,} $$

where P is the power requirement to automatic feeder, kW; I is the line current strength, Amperes; V is the potential difference, Voltage; Cos θ is the power factor, equal 0.8.

The specific energy consumption (SEC) in kW kg^−1^ was calculated by using the following equation:

4 $$ SEC = \frac{P}{{\Pr_{actual} }}{,} $$

where SEC is the specific energy consumption, W kg^−1^.

### Total costs

The cost calculation based on the following parameters was also performed:

#### Fixed costs (Fc)

- Depreciation costs (D_c_):
  5 $$ D_{c} = \frac{{P_{d} - S_{r} }}{{L_{d} }}{,} $$
  where Dc is the depreciation cost, EGP (Egyptian pound) year^−1^ ($ = 15.63 EGP); P_d_ is the automatic feeder purchase price, 10,000 EGP; Sr is the salvage rate (0.1P_d_) EGP; Ld is the automatic feeder life, 5 years.
- Interest costs (In):
  6 $$ I_{n} = \frac{{P_{d} + S_{r} }}{2} \times {\text{i}}_{{\text{n}}} {,} $$
  where I_n_ is the interest, EGP year^−1^; i_n_ is the interest as compounded annually, decimal (0.12).
- Shelter, taxes and insurance costs (Si):
  Shelter, taxes and insurance costs were assumed to be 3% of the purchase price of the automatic feeder (P_m_).
  Then:
  7 $$ {\text{Fixed cost (EGP h}}^{{ - {1}}} {)} = {\text{Dc}} + {\text{In}} + {\text{S}}i{\text{ / hour of use per year}}{.} $$

#### Variable (operating) costs (V_c_)

- Repair and maintenance costs (R_m_):
  8 $$ Rm = 100{\text{ \% deprecation cost / hour of use per year}}{.} $$
- Energy costs (E):
  9 $$ E \, = {\text{ EC }} \times {\text{ EP,}} $$
  where E is the energy costs, EGP h^−1^; EC is the electrical energy consumption, kWh; EP is the energy price, 0.57 EGP kW^−1^.
- Labor costs (La):
  10 $$ L{\text{a}} = {\text{ Salary of one worker }} \times {\text{ No}}{\text{. of workers ,}} $$
  where La is the Labor costs, EGP h^−1^; Salary of one worker = 10 EGP h^−1^; No. of workers = 1.
  Then:
  11 $$ {\text{Variable costs (EGP h}}^{{ - {1}}} ) = {\text{ R}}_{{\text{m}}} \, + {\text{E }} + {\text{La }}{.} $$

#### Total costs (Tc)

12 $$ {\text{Total cost (EGP h}}^{{ - {1}}} {) } = {\text{ Fixed cost (EGP h}}^{{ - {1}}} {) } + {\text{ Variable cost (EGP h}}^{{ - {1}}} {) }{\text{.}} $$

### Statistical analysis

The statistical analysis for the data obtained was done according to Ref.^20^ and the treatments were compared using Least Significant Differences (LSD) test at 99% confidence level^21^ using MSTAT-C software.

## Results and discussions

### Automatic feeder productivity

**Figure 4:**
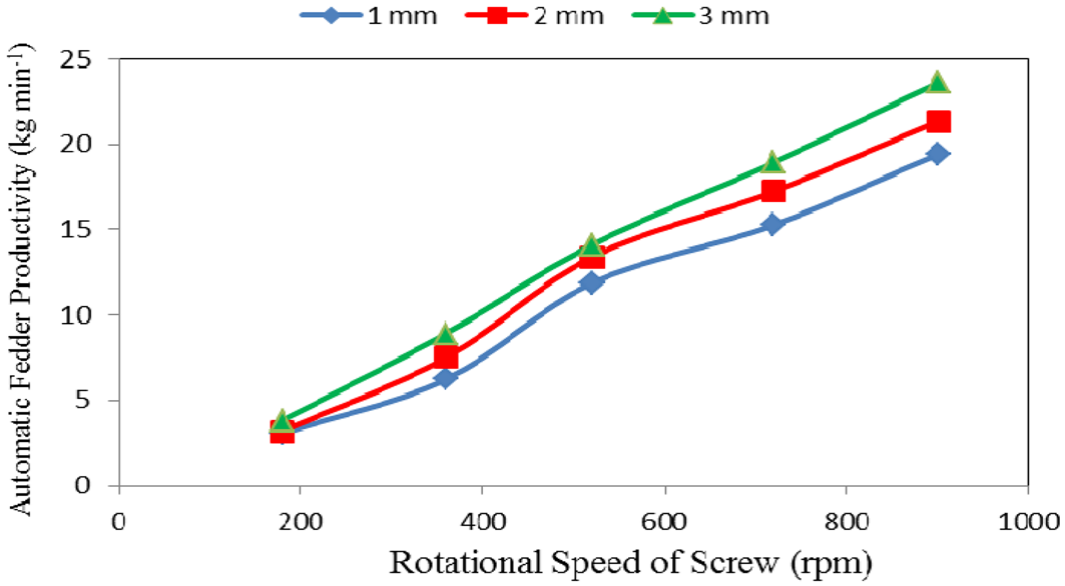
Automatic feeder productivity at different feed pellet sizes and rotational speeds of screw.

**Figure 5:**
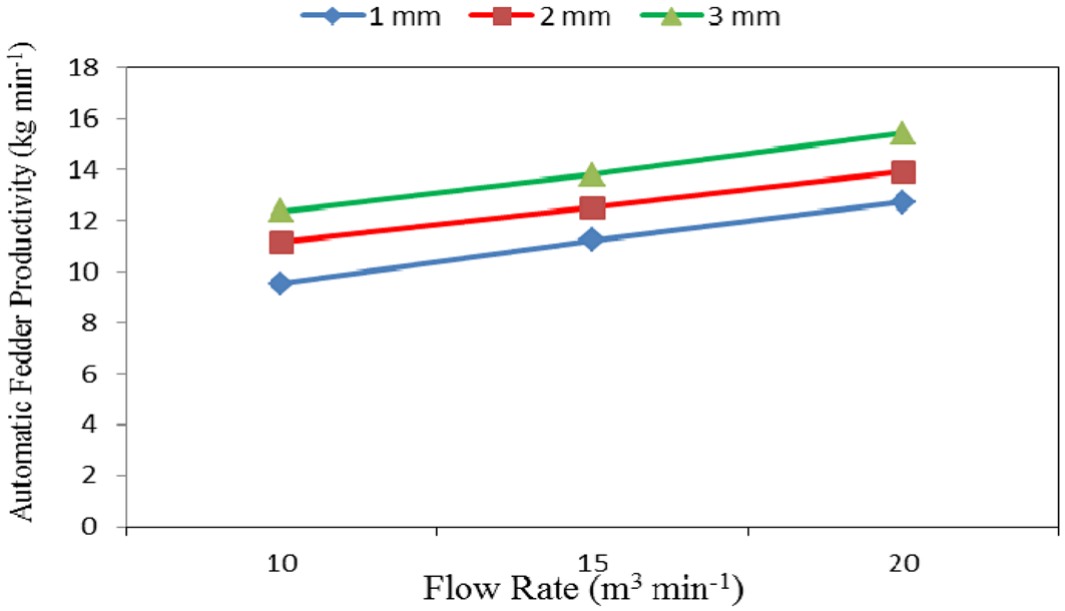
Automatic feeder productivity at different feed pellet sizes and air flow rates.

**Figure 6:**
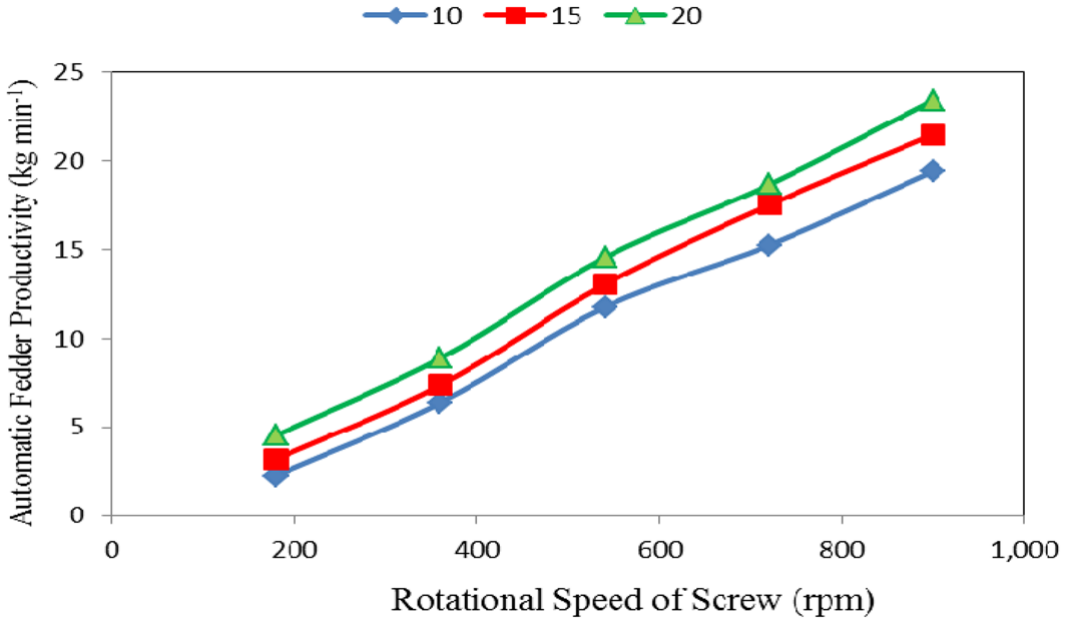
Automatic feeder productivity at different rotational speeds of screw and flow rates.

Table 1 and Figs. 4, 5 and 6 show the automatic feeder productivity as affected by the different feed pellets sizes (1, 2 and 3 mm), air flow rates (10, 15 and 20 m^3^ min^−1^) and rotational speeds of screw (180, 360, 540, 720 and 900 rpm). The results indicate that the automatic feeder productivity increases with increasing feed pellets size, air flow rate and rotational speed of screw. It indicates that when the feed pellets size increased from 1 to 3 mm, the automatic feeder productivity significantly increased from 11.16 to 13.87 (by 19.54%) kg min^−1^. It also indicates that when the air flow rate increased from 10 to 20 m^3^ min^−1^, the automatic feeder productivity significantly increased from 11.02 to 14.03 (by 21.45%) kg min^−1^, while the automatic feeder productivity significantly increased from 3.33 to 21.46 (by 84.48%) kg min^−1^ when the rotational speed of screw increased from 180 to 900 rpm.

**Table 1:** Automatic feeder productivity at different feed pellets sizes, air flow rates and rotational speeds of screw.

Feed pellets size, mm	Flow rate, m^3^ m^−1^	Rotational speed of screw, rpm					Mean
		180	360	540	720	900	
		Automatic feeder productivity, kg min^−1^					
1	10	2.02	5.13	10.19	13.26	17.04	9.53^a^
	15	2.96	6.01	11.95	15.89	19.36	11.23^b^
	20	4.15	7.55	13.45	16.65	21.86	12.73^c^
	Mean	3.04^a^	6.23^ab^	11.86^c^	15.27^d^	19.42^e^	
2	10	2.18	6.36	12.31	15.42	19.52	11.16^b^
	15	3.01	7.36	13.23	17.43	21.53	12.51^cd^
	20	4.3	8.77	14.63	18.87	23.02	13.92^d^
	Mean	3.16^a^	7.50^b^	13.39^cd^	17.24^e^	21.36^ef^	
3	10	2.57	7.67	12.78	17.07	21.76	12.37^bc^
	15	3.61	8.73	13.91	19.25	23.61	13.82^cd^
	20	5.18	10.37	15.6	20.51	25.48	15.43^e^
	Mean	3.79^a^	8.92^c^	14.10^d^	18.94^e^	23.62^g^	
Mean of size (A)	11.16^a^		12.53^a^		13.87^b^		
Mean of flow rate (B)	11.02^a^		12.52^b^		14.03^c^		
Mean of speed (C)	3.33^a^	7.55^b^		13.12^c^	17.15^d^	21.46^e^	
LSD at 0.05	A	B	C	AB	AC	BC	ABC
	1.37	1.41	2.29	2.30	1.35	2.45	N.S.

Superscripts letters mean significantly between the treatments (statistical analysis).

It could be noticed that increasing the feed pellets size from 1 to 3 mm, tends to increase the automatic feeder productivity from 3.04 to 3.79, 6.23 to 8.92, 11.86 to 14.10, 15.27 to 18.94 and 19.42 to 23.62 kg min^−1^ at 180, 360, 540, 720 and 900 rpm rotational speed of screw, respectively. The results also indicate that the automatic feeder productivity increased from 3.04 to 19.42, 3.16 to 21.36 and 3.79 to 23.62 kg min^−1^ at 1, 2 and 3 mm feed pellets sizes, respectively when the rotational speed of screw increased from 180 to 900 rpm as shown in Fig. 4.

From statistical analysis, there were no significant different between feed pellets sizes 1 and 2 on the automatic feeder productivity, meanwhile, there were significant differences between feed pellets size 3 and sizes 1 and 2 on the productivity. Regarding the effect of air flow rate, there were significant differences between air flow rates on the automatic feeder productivity, the same trend was happened with the effect of rotational speed of screw on productivity. The analysis showed also that the interaction between both ABC was non-significant. On the other hand, the interaction between the effect of both AB, AC and BC on the data was significant as shown in Table 1.

Regarding the effect of feed pellet size and air flow rate on the automatic feeder productivity, the results indicate that the automatic feeder productivity increases with increasing the feed pellets size and flow rate. It increased from 9.53 to 12.37, 11.23 to 13.82 and 12.73 to 15.43 kg min^−1^ for 10, 15 and 20 m^3^ min^−1^ air flow rate, respectively, when the feed pellets size increased from 1 to 3 mm. The results also indicate that the automatic feeder productivity increased from 9.53 to 12.73, 11.16 to 13.92 and 12.37 to 15.43 kg min^−1^ at 1, 2 and 3 mm feed pellets size, respectively, when the air flow rate increased from 10 to 20 m^3^ min^−1^ as shown in Fig. 5.

The results also indicate that the automatic feeder productivity increased from 2.26 to 4.54, 6.39 to 8.90, 11.76 to 14.56, 15.25 to 18.68 and 19.44 to 23.45 kg min^−1^ at 180, 360, 540, 720 and 900 rpm rotational speed of screw, respectively, when the air flow rate increased from 10 to 20 m^3^ min^−1^. The results also indicate that the automatic feeder productivity increased from 2.26 to 19.44, 3.19 to 21.50 and 4.54 to 23.45 kg min^−1^ at 10, 15 and 20 m^3^ min^−1^ air flow rate, respectively, when the rotational speed of screw increased from 180 to 900 rpm as shown in Fig. 6.

Multiple regression analysis was carried out to obtain a relationship between the automatic feeder productivity as dependent variable and different of feed pellets size, air flow rate and rotational speed of screw as independent variables. The best fit for this relationship is presented in the following equation:-

13 $$ \Pr_{actual} = - 8.457 + 1.354PS + 0.301FR + 0.025RS{\text{ R}}^{{2}} = 0.98{ ,} $$

where PS is the feed pellets size, mm; FR is the air flow rate, m^3^ min^−1^; RS is the rotational speed of screw, rpm.

This equation could be applied in the range of 1 to 3 mm feed pellets size, 10 to 20 m^3^ min^−1^ air flow rate and from 180 to 900 rpm of rotational speed of screw.

### Automatic feeder efficiency

**Figure 7:**
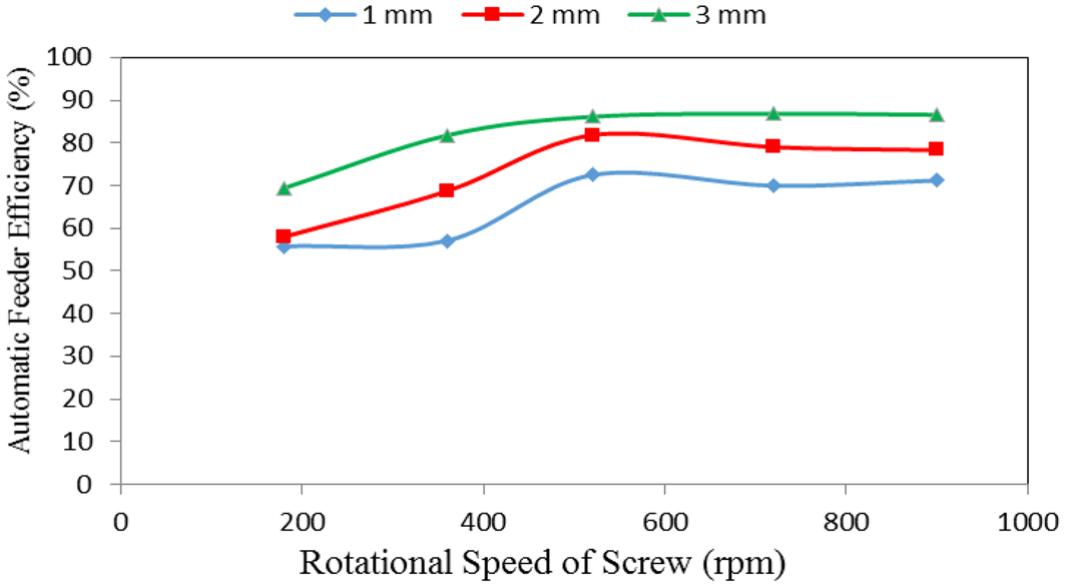
Automatic feeder efficiency at different feed pellet sizes and rotational speeds of screw.

**Figure 8:**
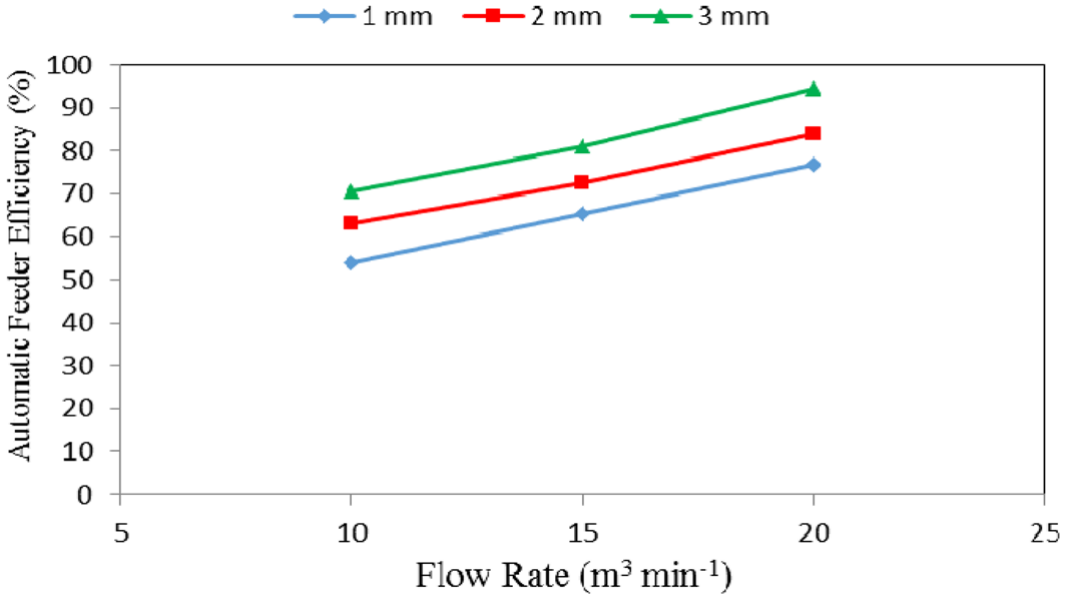
Automatic feeder efficiency at different feed pellet sizes and air flow rates.

**Figure 9:**
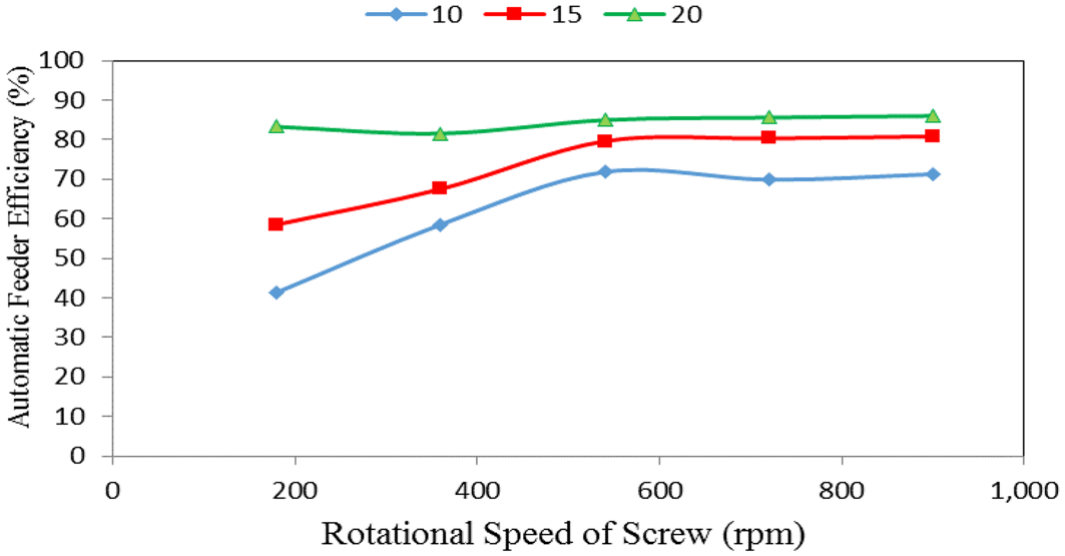
Automatic feeder efficiency at different rotational speeds of screw and air flow rates.

Table 2, Figs. 7, 8 and 9 show the automatic feeder efficiency as affected by the different feed pellets sizes (1, 2 and 3 mm), air flow rates (10, 15 and 20 m^3^ min^−1^) and rotational speeds of screw (180, 360, 540, 720 and 900 rpm). The results indicate that, when the feed pellets size increased from 1 to 3 mm, the automatic feeder efficiency significantly increased from 65.30 to 82.14 (by 20.50%) %. It also indicates that when the air flow rate increased from 10 to 20 m^3^ min^−1^, the automatic feeder efficiency significantly increased from 62.58 to 85.07 (by 26.44%) %, while the automatic feeder efficiency significantly increased from 61.58 to 78.69 (by 21.74%) % when the rotational speed of screw increased from 180 to 900 rpm.

**Table 2:** Automatic feeder efficiency at different feed pellets sizes, air flow rates and rotational speeds of screw.

Feed pellets size, mm	Flow rate, m^3^ m^−1^	Rotational speed of screw, rpm					Mean
		180	360	540	720	900	
		Efficiency of automatic feeder, %					
1	10	37.03	47.02	62.26	60.76	62.47	53.91^a^
	15	54.26	55.08	73.01	72.82	70.97	65.23^b^
	20	76.07	69.20	82.18	76.30	80.14	76.78^c^
	Mean	55.79^a^	57.10^a^	72.48^b^	69.96^b^	71.19^b^	
2	10	39.96	58.29	75.21	70.66	71.56	63.14^b^
	15	55.17	67.45	80.83	79.87	78.93	72.45^bc^
	20	78.82	80.38	89.39	86.47	84.39	83.89^d^
	Mean	57.98^a^	68.71^b^	81.81^c^	79.00^b^	78.29^b^	
3	10	47.11	70.29	78.08	78.22	79.77	70.69^b^
	15	66.17	80.01	84.99	88.21	86.55	81.19^cd^
	20	94.95	95.04	95.32	93.99	93.41	94.54^e^
	Mean	69.41^b^	81.78^bc^	86.13^c^	86.81^c^	86.58^c^	
Mean of size (A)	65.30^a^			73.16^b^		82.14^c^	
Mean of flow rate (B)	62.58^a^			72.95^b^		85.07^c^	
Mean of speed (C)	61.06^a^	69.20^b^		80.14^c^	78.59^c^		78.69^c^
LSD at 0.05	A	B	C	AB	AC	BC	ABC
	5.28	6.03	6.57	7.11	8.62	6.95	N.S

Superscripts letters mean significantly between the treatments (statistical analysis).

It could be noticed that increasing the feed pellets size from 1 to 3 mm, tends to increase the automatic feeder efficiency from 55.79 to 69.41, 57.10 to 81.78, 72.48 to 86.13, 69.96 to 86.81 and 71.19 to 86.58% at 180, 360, 540, 720 and 900 rpm rotational speed of screw, respectively. The results also indicate that the automatic feeder efficiency increased from 55.79 to 71.19, 57.98 to 78.29 and 69.41 to 86.58% at 1, 2 and 3 mm feed pellets sizes, respectively when the rotational speed of screw increased from 180 to 900 rpm as shown in Fig. 7.

The statistical analysis showed that the differences between the obtained data of automatic feeder efficiency due to the effect of feed pellets size (A) and air flow rate (B) were significant. Regarding the effect of rotational speed of screw, there were significant differences between rotational speeds of screw 1, 2 and 3, meanwhile, there were no significant differences between rotational speeds of screw 3, 4 and 5. The analysis showed also that the interaction between both ABC was non-significant. On the other hand, the interaction between the effect of both AB, AC and BC on the data was significant as shown in Table 2.

Regarding the effect of feed pellet size and air flow rate on the automatic feeder productivity, the results indicate that the automatic feeder efficiency increases with increasing the feed pellets size and flow rate. It increased from 53.91 to 70.69, 65.23 to 81.19 and 76.78 to 94.54% for 10, 15 and 20 m^3^ min^−1^ air flow rate, respectively, when the feed pellets size increased from 1 to 3 mm. The results also indicate that the automatic feeder efficiency increased from 53.91 to 76.78, 63.14 to 83.89 and 70.69 to 94.54% at 1, 2 and 3 mm feed pellets size, respectively, when the air flow rate increased from 10 to 20 m^3^ min^−1^ as shown in Fig. 8.

The results also indicate that the automatic feeder efficiency increased from 41.37 to 83.28, 58.53 to 81.54, 71.85 to 84.96, 69.88 to 85.59 and 71.27 to 85.98% at 180, 360, 540, 720 and 900 rpm rotational speed of screw, respectively, when the air flow rate increased from 10 to 20 m^3^ min^−1^. The results also indicate that the automatic feeder efficiency increased from 41.37 to 71.27, 58.53 to 80.82 and 83.28 to 85.98% at 10, 15 and 20 m^3^ min^−1^ air flow rate, respectively, when the rotational speed of screw increased from 180 to 900 rpm as shown in Fig. 9.

Increasing the parameters seams to increase the productivity but regarding the efficiency, results show that the efficiency increases with increasing this parameter at (540 rpm) started to be constant and 720–900 rpm decreased in all treatments under study (Figs. 7, 9). It is concluded that efficiency with the parameters increased, became constant and decreased.

Multiple regression analysis was carried out to obtain a relationship between the automatic feeder efficiency as dependent variable and different of feed pellets size, air flow rate and rotational speed of screw as independent variables. The best fit for this relationship is presented in the following equation:-

14 $$ \eta = 9.566 + 8.417PS + 2.249FR + 0.025RS{\text{ R}}^{{2}} = 0.89{ ,} $$

where this equation could be applied in the range of 1 to 3 mm feed pellets size, 10 to 20 m^3^ min^−1^ air flow rate and from 180 to 900 rpm of rotational speed of screw.

### Specific energy consumption

**Figure 10:**
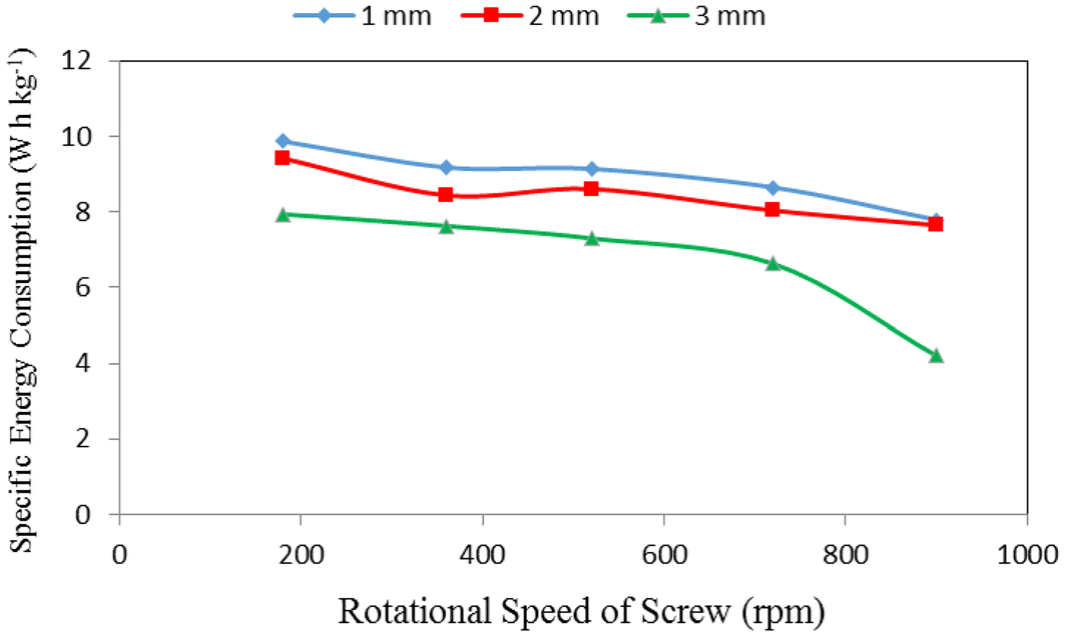
Specific energy consumption at different feed pellet sizes and rotational speeds of screw.

**Figure 11:**
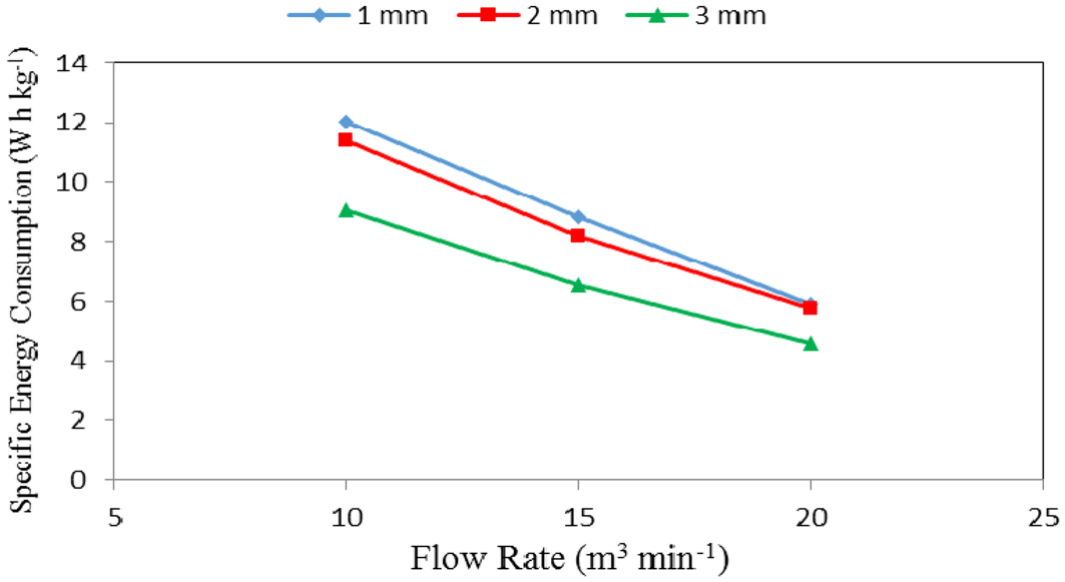
Specific energy consumption at different feed pellet sizes and air flow rates.

**Figure 12:**
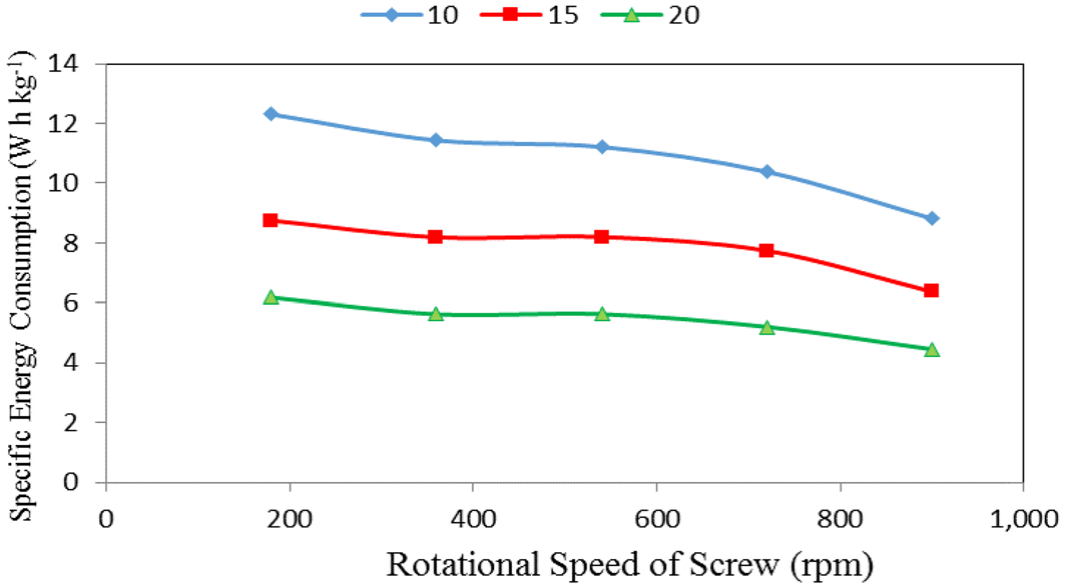
Specific energy consumption at different rotational speeds of screw and air flow rates.

Table 3, Figs. 10, 11 and 12 show the specific energy consumption of automatic feeder as affected by the different feed pellets sizes (1, 2 and 3 mm), air flow rates (10, 15 and 20 m^3^ min^−1^) and rotational speeds of screw (180, 360, 540, 720 and 900 rpm). The results indicate that the specific energy consumption of automatic feeder decreases with increasing feed pellets size, air flow rate and rotational speed of screw. It indicates that when the feed pellets size increased from 1 to 3 mm, the specific energy consumption of automatic feeder significantly decreased from 8.93 to 6.74 (by 24.52%) W h kg^−1^. It also indicates that when the air flow rate increased from 10 to 20 m^3^ min^−1^, the specific energy consumption of automatic feeder significantly decreased from 10.83 to 5.42 (by 49.95%) W h kg^−1^, while the specific energy consumption significantly decreased from 9.08 to 6.55 (by 27.86%) W h kg^−1^ when the rotational speed of screw increased from 180 to 900 rpm.

**Table 3:** Specific energy consumption at different feed pellets sizes, air flow rates and rotational speeds of screw.

Feed pellets size, mm	Flow rate, m^3^ min^−1^	Rotational speed of screw, rpm					Mean
		180	360	540	720	900	
		Specific energy consumption, W h kg^−1^					
1	10	13.6	12.57	12.19	11.37	10.52	12.05^c^
	15	9.34	9.01	9.14	8.98	7.59	8.81^b^
	20	6.68	5.97	6.09	5.61	5.27	5.92^a^
	Mean	9.87^bc^	9.18^b^	9.14^b^	8.65^b^	7.79^b^	
2	10	12.60	11.46	11.60	10.89	10.29	11.37^c^
	15	9.21	8.13	8.38	7.76	7.42	8.18^ab^
	20	6.46	5.74	5.85	5.48	5.23	5.75^a^
	Mean	9.42^b^	8.44^b^	8.61^b^	8.04^b^	7.65^b^	
3	10	10.72	10.27	9.85	8.88	5.63	9.07^b^
	15	7.71	7.43	7.08	6.48	4.09	6.56^a^
	20	5.39	5.18	4.96	4.53	2.87	4.59^a^
	Mean	7.94^b^	7.63^b^	7.30^b^	6.63^b^	4.20^a^	
Mean of size (A)	8.93^b^		8.43^b^		6.74^a^		
Mean of time (B)	10.83^c^		7.85^b^		5.42^a^		
Mean of speed (C)	9.08^c^	8.42^bc^		8.35^b^	7.78^b^		6.55^a^
LSD at 0.05	A	B	C	AB	AC	BC	ABC
	1.01	2.13	1.14	2.15	2.47	2.07	N.S

Superscripts letters mean significantly between the treatments (statistical analysis).

It could be noticed that increasing the feed pellets size from 1 to 3 mm, tends to decrease the specific energy consumption from 9.87 to 7.94, 9.18 to 7.63, 9.14 to 7.30, 8.65 to 6.63 and 7.79 to 4.20 W h kg^−1^ at 180, 360, 540, 720 and 900 rpm rotational speed of screw, respectively. The results also indicate that the specific energy consumption decreased from 9.87 to 7.79, 9.42 to 7.65 and 7.94 to 4.20 W h kg^−1^ at 1, 2 and 3 mm feed pellets sizes, respectively when the rotational speed of screw increased from 180 to 900 rpm as shown in Fig. 10.

From statistical analysis, there were no significant differences between feed pellets sizes 1 and 2 on the specific energy consumption, meanwhile, there were significant differences between feed pellets size 3 and 1 and 2 on the specific energy consumption. Regarding the effect of air flow rate, there were significant differences between air flow rates and specific energy consumption. Regarding the effect of rotational speed of screw, there were significant differences between rotational speeds of screw 1, 2, 4 and 5 on the specific energy consumption, meanwhile, there were no significant differences between rotational speeds of screw 2 and 3 on the specific energy consumption. The analysis showed also that the interaction between both ABC was non-significant. On the other hand, the interaction between the effect of both AB, AC and BC on the data was significant as shown in Table 3.

Regarding the effect of feed pellet size and air flow rate on the specific energy consumption, the results indicate that the specific energy consumption decreases with increasing the feed pellets size and flow rate. It decreased from 12.05 to 9.07, 8.81 to 6.56 and 5.92 to 4.59 W h kg^−1^ for 10, 15 and 20 m^3^ min^−1^ air flow rate, respectively, when the feed pellets size increased from 1 to 3 mm. The results also indicate that the specific energy consumption decreased 12.05 to 5.92, 11.37 to 5.75 and 9.07 to 4.59 W h kg^−1^ at 1, 2 and 3 mm feed pellets size, respectively, when the air flow rate increased from 10 to 20 m^3^ min^−1^ as shown in Fig. 11.

The results also indicate that the specific energy consumption decreased from 12.31 to 6.18, 11.43 to 5.63, 11.21 to 5.63, 10.38 to 5.21 and 8.81 to 4.46 W h kg^−1^ at 180, 360, 540, 720 and 900 rpm rotational speed of screw, respectively, when the air flow rate increased from 10 to 20 m^3^ min^−1^. The results also indicate that the specific energy consumption decreased from 12.31 to 8.81, 8.75 to 6.37 and 6.18 to 4.46 W h kg^−1^ at 10, 15 and 20 m^3^ min^−1^ air flow rate, respectively, when the rotational speed of screw increased from 180 to 900 rpm as shown Fig. 12.

Multiple regression analysis was carried out to obtain a relationship between the specific energy consumption of automatic feeder as dependent variable and different of feed pellets size, air flow rate and rotational speed of screw as independent variables. The best fit for this relationship is presented in the following equation:-

15 $$ SEC = 20.045 - 1.095PS - 0.541FR - 0.003RS{\text{ R}}^{{2}} = 0.92 \, {.} $$

This equation could be applied in the range of 1 to 3 mm feed pellets size, 10 to 20 m^3^ min^−1^ air flow rate and from 180 to 900 rpm of rotational speed of screw.

### Total costs of automatic feeder

**Figure 13:**
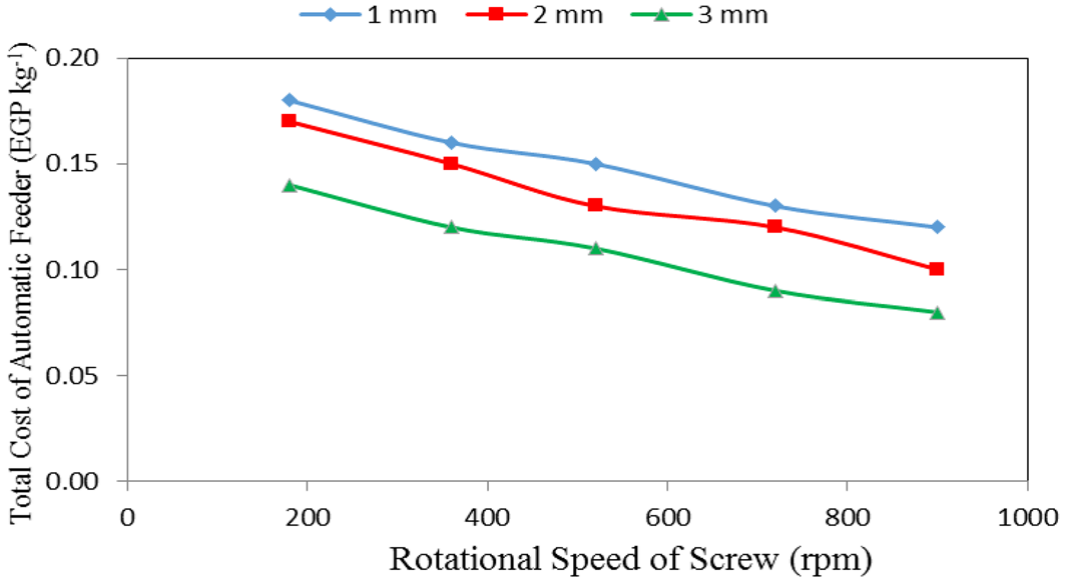
Total cost of automatic feeder at different feed pellet sizes and rotational speeds of screw.

**Figure 14:**
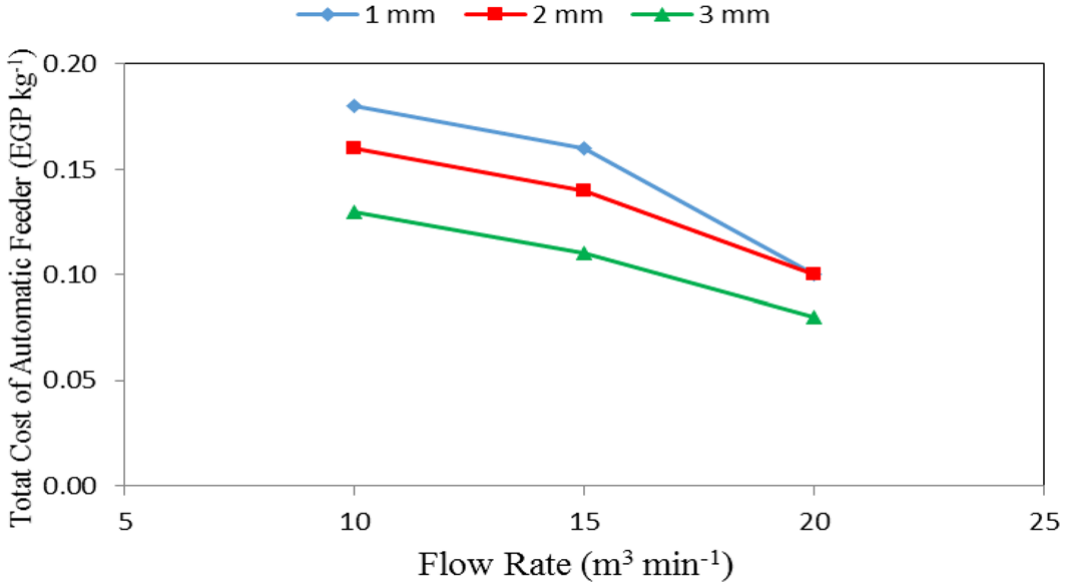
Total cost of automatic feeder at different feed pellet sizes and air flow rates.

**Figure 15:**
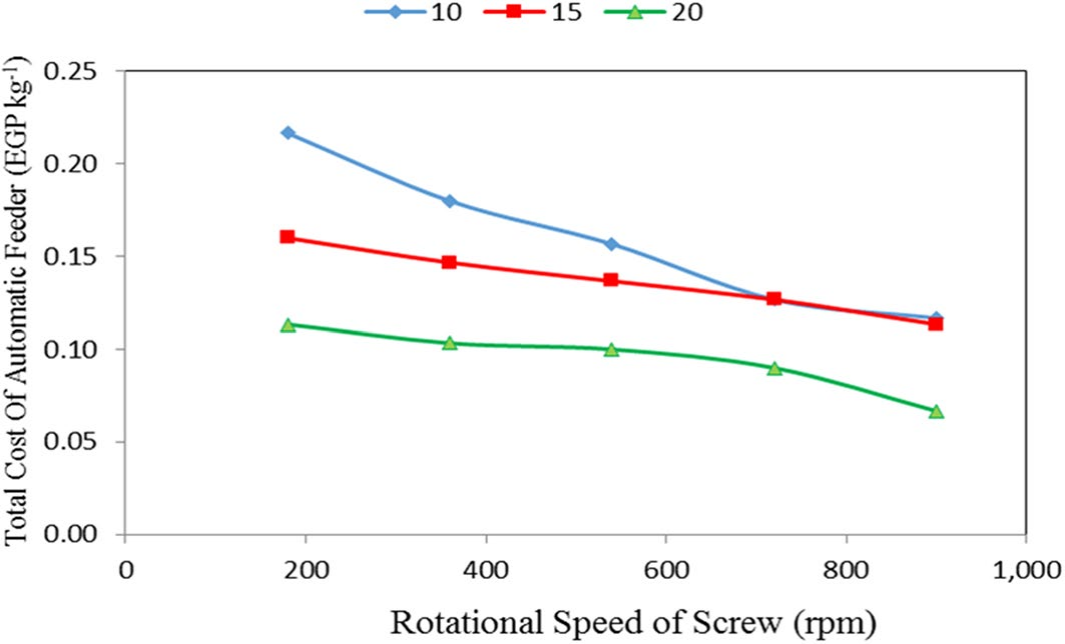
Total cost of automatic feeder at different rotational speeds of screw and air flow rate.

Table 4, Figs. 13, 14 and 15 show the total cost of automatic feeder as affected by the different feed pellets sizes (1, 2 and 3 mm), air flow rates (10, 15 and 20 m^3^ min^−1^) and rotational speeds of screw (180, 360, 540, 720 and 900 rpm). The results indicate that the total cost of automatic feeder decreases with increasing feed pellets size, flow rate and rotational speed of screw. It indicates that when the feed pellets size increased from 1 to 3 mm, the total cost of automatic feeder significantly decreased from 0.15 to 0.11 (by 26.27%) EGP kg^−1^. It also indicates that when the air flow rate increased from 10 to 20 m^3^ min^−1^, the total cost of automatic feeder significantly decreased from 0.16 to 0.09 (by 43.75%) EGP kg^−1^, while the total cost of automatic feeder significantly decreased from 0.16 to 0.10 (by 37.50%) EGP kg^−1^ when the rotational speed of screw increased from 180 to 900 rpm.

**Table 4:** Total cost of automatic feeder at different feed pellets sizes, air flow rate and rotational speeds of screw.

Feed pellets size, mm	Flow rate, m^3^ min^−1^	Rotational speed of screw, rpm					Mean
		180	360	540	720	900	
		Total cost of automatic feeder, EGP kg^−1^					
1	10	0.24	0.21	0.18	0.15	0.14	0.18
	15	0.17	0.17	0.16	0.15	0.14	0.16
	20	0.12	0.11	0.11	0.10	0.07	0.10
	Mean	0.18	0.16	0.15	0.13	0.12	
2	10	0.23	0.18	0.16	0.13	0.12	0.16
	15	0.17	0.15	0.14	0.13	0.12	0.14
	20	0.12	0.11	0.10	0.09	0.07	0.10
	Mean	0.17	0.15	0.13	0.12	0.10	
3	10	0.18	0.15	0.13	0.10	0.09	0.13
	15	0.14	0.12	0.11	0.10	0.08	0.11
	20	0.1	0.09	0.09	0.08	0.06	0.08
	Mean	0.14	0.12	0.11	0.09	0.08	
Mean of size (A)	0.15^ab^		0.13^a^			0.11^a^	
Mean of time (B)	0.16^b^		0.14^b^			0.09^a^	
Mean of speed (C)	0.16^bc^	0.14^b^		0.13^ab^	0.11^a^		0.10^a^
LSD at 0.05	A	B	C	AB	AC	BC	ABC
	0.03	0.03	0.02	N.S.	N.S.	N.S	N.S.

Superscripts letters mean significantly between the treatments (statistical analysis).

It could be noticed that increasing the feed pellets size from 1 to 3 mm, tends to decrease the total cost of automatic feeder from 0.18 to 0.14, 0.16 to 0.12, 0.15 to 0.11, 0.13 to 0.09 and 0.12 to 0.08 EGP kg^−1^ at 180, 360, 540, 720 and 900 rpm rotational speed of screw, respectively. The results also indicate that the total cost of automatic feeder decreased from 0.18 to 0.12, 0.17 to 0.10 and 0.14 to 0.08 EGP kg^−1^ at 1, 2 and 3 mm feed pellets sizes, respectively when the rotational speed of screw increased from 180 to 900 rpm as shown in Fig. 13.

From statistical analysis, there were no significant differences between feed pellets sizes 1 and 2 on the total cost of automatic feeder, meanwhile, there were significant differences between feed pellets size 3 and 1 and 2 on the total cost of automatic feeder. Regarding the effect of air flow rate, there were significant differences between air flow rates and specific energy consumption. Regarding the effect of rotational speed of screw, there were no significant differences between rotational speeds of screw 1 and 2, also 3 and 4 on the total cost of automatic feeder, meanwhile, there were significant differences between rotational speeds of screw 2 and 3 on the total cost of automatic feeder.

Regarding the effect of feed pellet size and flow rate on the total cost of automatic feeder, the results indicate that the total cost of automatic feeder decreases with increasing the feed pellets size and air flow rate. It decreased from 0.18 to 0.13, 0.16 to 0.11 and 0.10 to 0.08 EGP kg^−1^ for 10, 15 and 20 m^3^ min^−1^ air flow rate, respectively, when the feed pellets size increased from 1 to 3 mm. The results also indicate that the total cost of automatic feeder decreased from 0.18 to 0.10, 0.16 to 0.10 and 0.13 to 0.08 EGP kg^−1^ at 1, 2 and 3 mm feed pellets size, respectively, when the air flow rate increased from 10 to 20 m^3^ min^−1^ as shown in Fig. 14.

The results also indicate that the total cost of automatic feeder decreased from 0.22 to 0.11, 0.18 to 0.10, 0.16 to 0.10, 0.13 to 0.09 and 0.12 to 0.07 EGP kg^−1^ at 180, 360, 540, 720 and 900 rpm rotational speed of screw, respectively, when the air flow rate increased from 10 to 20 m^3^ min^−1^. The results also indicate that the total cost of automatic feeder decreased from 0.22 to 0.12, 0.16 to 0.11 and 0.11 to 0.07 EGP kg^−1^ for 10, 15 and 20 m^3^ min^−1^ air flow rate, respectively, when the rotational speed of screw increased from 180 to 900 rpm as shown in Fig. 15.

Multiple regression analysis was carried out to obtain a relationship between the total costs of automatic feeder as dependent variable and different of feed pellets size, air flow rate and rotational speed of screw as independent variables. The best fit for this relationship is presented in the following equation:

16 $$ TC = 0.315 - 0.020PS - 0.006FR - 8.8 \times 10^{ - 5} RS{\text{ R}}^{{2}} = 0.87{,} $$

where: TC is the total cost of automatic feeder, EGP kg^−1^.

This equation could be applied in the range of 1 to 3 mm feed pellets size, 10 to 20 m^3^ min^−1^ air flow rate and from 180 to 900 rpm of rotational speed of screw.

## Conclusion

Accuracy in feeding quantities and timing is very effective in fish growth rate and production. From this study. It is concluded that, the feeder which was manufactured using local raw materials at the workshop of Agricultural and Bio-Systems Engineering Department, Faculty of Agriculture Moshtohor, Benha University, Egypt was tested and evaluated, where, its productivity ranged from 3.33 to 21.46 kg, efficiency ranged from 61.58 to 85.07% and the specific energy consumption ranged from 5.42 to 10.83 W h kg^−1^. Finally, the total cost ranged from 0.09 to 0.16 EGP kg^−1^ ($ = 15.63 EGP) under different operational conditions which is considered very cheep. This feeder is cost effective tool which helps fish producer to be able to feed the fish without delaying or variations in distribution of feeds while in turn improve the quality of fish production. Also, it is saved time, easy, effort and cost-effective and it could be working you are away from the fish farm.
